# Thermoplastic Pultrusion: A Review

**DOI:** 10.3390/polym13020180

**Published:** 2021-01-06

**Authors:** Kirill Minchenkov, Alexander Vedernikov, Alexander Safonov, Iskander Akhatov

**Affiliations:** Skolkovo Institute of Science and Technology, Center for Design, Manufacturing and Materials, 30/1 Bolshoi Boulevard, 121205 Moscow, Russia; kirill.minchenkov@skoltech.ru (K.M.); aleksandr.vedernikov@skoltech.ru (A.V.); i.akhatov@skoltech.ru (I.A.)

**Keywords:** thermoplastic pultrusion, thermoplastic composites, fiber-reinforced materials

## Abstract

Pultrusion is one of the most efficient methods of producing polymer composite structures with a constant cross-section. Pultruded profiles are widely used in bridge construction, transportation industry, energy sector, and civil and architectural engineering. However, in spite of the many advantages thermoplastic composites have over the thermoset ones, the thermoplastic pultrusion market demonstrates significantly lower production volumes as compared to those of the thermoset one. Examining the thermoplastic pultrusion processes, raw materials, mechanical properties of thermoplastic composites, process simulation techniques, patents, and applications of thermoplastic pultrusion, this overview aims to analyze the existing gap between thermoset and thermoplastic pultrusions in order to promote the development of the latter one. Therefore, observing thermoplastic pultrusion from a new perspective, we intend to identify current shortcomings and issues, and to propose future research and application directions.

## 1. Introduction

Today, polymer composite materials have found wide application in various industries [1,2]. This was made possible by extensive studies conducted over the last 70 years [3,4]. The popularity of composite materials results from their properties, such as high specific strength and stiffness [5,6,7,8]; improved durability [9,10]; high fatigue [11,12], chemical [13,14], and corrosion resistance [15,16,17]; and ease of transportation and assembly [18,19,20] of composite structures. Composite materials are produced by various processes, e.g., autoclave molding, resin transfer molding, compression molding, filament winding, and pultrusion [21,22]. Pultrusion is a process where a pack of reinforcement fibers impregnated by resin is pulled through a heated die block, where the polymerization process takes place [23]. This method allows fabrication of products having constant cross-section [24,25]. The advantages of pultrusion over other composite manufacturing processes are its high production rate of up to 5 m/min [1], higher efficiency [26,27] and low costs [28,29] of production, and the ability to produce profiles of virtually indefinite length [30]. There are thermoplastic and thermoset matrix-based composites [31,32]. Thermosets are nonmelting polymers obtained during chemical reaction (polymerization) between a resin and a hardener, while thermoplastic composites can change their state and melt under heating. Fiber reinforcement is impregnated by hot melt thermoplastic polymer; then, after cooling, the part is ready for use. Compared to thermosetting composites, thermoplastic composites have higher impact toughness [33,34,35,36], are faster to produce [37,38], have higher service temperatures [39], can be joined by welding [40,41,42,43], have less environmental impact [44,45,46], and can be recycled [47,48,49,50]; their source materials have virtually unlimited shelf life [51,52,53,54,55,56,57]. Pultruded thermoplastic profiles are used in various structures and sectors, such as vehicles [58,59,60,61,62,63] and aircrafts construction [64,65,66], aerospace [67,68,69] and civil engineering [70,71,72,73], energy systems [74], restoration of deteriorated structures [75], marine applications [76,77,78,79], oil and gas industries [80], electromagnetic interference shielding elements [81,82], window profiles [83], pipes [84,85], rebars [86,87] and rods [88,89,90,91].

Today, the number of studies and publications in the field of thermoset pultrusion is an order of magnitude larger than the number of those in thermoplastic pultrusion, although advantages offered by thermoplastic composites provide enough reason for deeper study. From industrial point of view, it is worth noticing that, for instance, Fiberline Composites A/S, being one of the largest companies in the pultrusion manufacturing and the world’s biggest web-shop of fiberglass profiles [92], has numerous types of pultruded structural profiles available for purchase with all of them being thermoset ones. Several questions arise in this connection. Why are there no thermoplastic profiles available for the customers to purchase, despite their numerous advantages? Why is there a well-developed market for thermosetting profiles and almost no market for thermoplastic ones?

Currently, there is no review on thermoplastic pultrusion. By analyzing the thermoplastic pultrusion process from different perspectives (technology, raw materials, properties, numerical modeling, applications, etc.) and recalling the main publications regarding thermoplastic pultrusion (scientific articles, patents), this article aims to understand why thermoplastic pultrusion failed to receive the attention and broad acceptance it deserves from the scientific and engineering community, as opposed to that of thermoset pultrusion. Exploring this issue, the authors intend to attract scientists’ attention and stimulate further developments in thermoplastic pultrusion. Section 2 describes the process of thermoplastic pultrusion and components of pultrusion machines, provides the classification of thermoplastic pultrusion, analyzes basic parameters of pultrusion process, and reviews patents registered and future research possibilities. Section 3 discusses raw materials used in manufacturing of thermoplastic composites, mechanical properties of the pultruded profiles, and promising areas for the further investigations. Section 4 reviews the methods of process modeling and discusses of possible directions for scientific work. Section 5 analyzes the possible applications of thermoplastic profiles, patents registered, and future research and application possibilities.

## 2. Thermoplastic Pultrusion and Process Parameters

**Figure 1:**
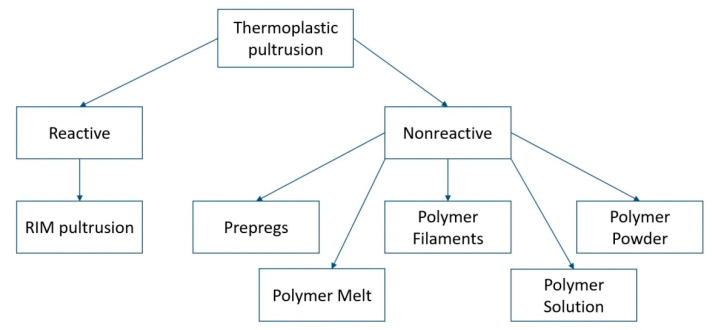
Thermoplastic pultrusion types.

Luisier et al. were the first to propose the classification of thermoplastic pultrusion processes in two groups [93]. The first is nonreactive thermoplastic pultrusion where the process is based on the already polymerized materials, as opposed to the second one—reactive thermoplastic pultrusion where thermoplastic is polymerized during chemical reaction between thermoplastic resin and catalyst/activator, with simultaneous impregnation of fiber reinforcement (Figure 1) [93].

**Figure 2:**
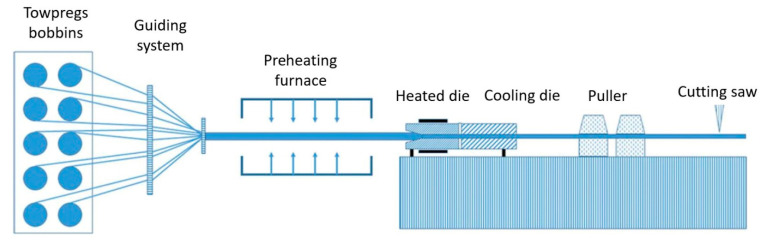
Schematic diagram of the nonreactive thermoplastic pultrusion line.

A nonreactive pultrusion machine for thermoplastic composites consists of towpregs bobbins, guiding system, preheating chamber (preheater), heated forming die, cooling die, puller, and a cutting saw (Figure 2) [30]. Pretreated fibers, blended intimately with thermoplastics at the filament level at certain ratio, are fed into the guiding system in order to prevent their entanglement and to distribute fibers over the whole section of the profile. Collimated fibers are then fed into a preheater and heated to a temperature above the melting point of the thermoplastic in order to reduce the time the reinforcement stays in the heated die block and to ensure uniform impregnation of reinforcement. Various types of heating systems can be used in the process, such as convective [94], infrared [95,96,97], contact [98], and microwave [99]. In practice, contact heating systems demonstrate higher efficiency, as compared to convective ones [94]. After exiting the preheating chamber, uniformly heated material enters the heated die block where the melting of thermoplastic takes place and the profile assumes its final shape. In order to accelerate the consolidation of the polymer, the formed profile is fed into the cooling die where it is cooled to the near-ambient temperature. At the last stage the profile is cut to the required lengths with a flying saw [100].

Nonreactive pultrusion machines can also incorporate braiding appliances [101,102]. Bechtold et al. [94] have successfully combined pultrusion and braiding, producing the profile with additional external reinforcement. The Daimler AG company has investigated and patented the braided pultrusion machine allowing fabrication of hollow profiles [103,104,105,106,107]. Memon and Nakai [108] used the combination of pultrusion and braiding to fabricate pipes reinforced with jute fiber. They tested various pulling speeds, temperatures, and pulling forces, and also investigated the influence of braiding parameters such as the braiding angle, the gap between braiding yarns, and the filling ratio.

From the industrial point of view, it is worth noting the knowledge that was developed in the leading countries with patents on thermoplastic pultrusion: USA, China, Germany, and France. Engineers developed various techniques to control the tension of the filaments [109,110], the pressure in the die [111,112], and the size of the die cavity [113,114]. Different techniques of fiber impregnation [115,116,117,118,119,120] and material feeding, such as sheet feeding of fibers and thermoplastics [121] and individual fiber feeding [122,123], were developed. Pultrusion is normally used to create profiles of constant cross-section; however, engineers from Boeing and the Phillips Petroleum Company modified the mechanics of the process, making it possible to produce profiles of variable cross-section, either by using multiple dies [124,125] or by modifying the die system [126,127,128,129,130].

**Figure 3:**
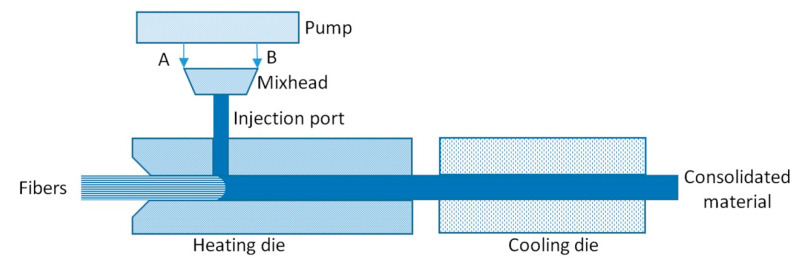
Scheme of the reaction injection molding (RIM) pultrusion die block.

The combination of pultrusion and reaction injection molding (RIM) resulted in development of the RIM pultrusion (reactive pultrusion) process similar to the combination of thermoset pultrusion and injection molding [131,132,133,134,135,136], patented by Industrial Technology Research Institute in 1993 [137]. The main difference between the reactive and nonreactive pultrusion processes is the design of the heated die block. In the reactive pultrusion process, preheated unimpregnated fiber is fed into the heated die block where fiber impregnation and polymerization of matrix take place (in situ polymerization), and the polymerized matrix has properties of thermoplastic melt [40]. The following polymers are typically used in the RIM pultrusion: polycarbonates (PC), polyesters (PE), polyurethanes (PU), polymethylmethacrylates (PMMA), and polyamides (PA) (in particular, PA-6 synthesized from ε-caprolactam (ε-CL) monomer) [138]. Figure 3 shows a schematic illustration of the RIM pultrusion die block [139].

The important advantage of reactive pultrusion lies in the low viscosity of thermoplastic resin solution as opposed to thermoplastic polymers, which improves and accelerates impregnation and, in turn, increases production rate. The important factor is the rate of polymerization, as it can take 1 to 60 min for polymerization to complete, depending on the temperature and monomer-to-activator ratio [140,141,142].

Further in the chapter we will discuss the parameters of the nonreactive thermoplastic pultrusion process, such as preheater temperature, temperature and geometry of the heated die, pressure inside the heated die, cooling die temperature, pulling speed, and pulling force, and their influence on the production process. We will not limit the discussion to the description of the process, but will also include a brief overview of articles investigating a particular manufacturing parameter from a scientific point of view, and, finally, note promising areas for future research.

### 2.1. Preheater Temperature

The aim of a preheating system analysis is to find the optimum temperature that would allow maximum increase in production rate without compromising the performance of the profiles produced. Increase in pulling speed reduces the time a material stays in the preheating chamber and, thus, requires the use of more efficient heating techniques. In 1997, Carlsson and Astrom [95] suggested that a preheater should meet the following requirements: the heating should be noncontact (to prevent melting of thermoplastic), continuous (to prevent overheating and degradation of the material), and uniform (to prevent temperature differences in a material).

However, as was shown in practice, the use of a contact preheater allows engineers to speed up the process, increase heating efficiency, and improve the shear strength of the material [94]. The optimum preheater temperature is assumed to be close to the melting temperature of a thermoplastic, in spite of the fact that high preheating temperature reduces the viscosity and drag while reducing the probability of fiber breakage [143]. On the other hand, preheating temperature that exceeds the thermoplastic melting temperature may cause the partial loss of material and increased void content, especially when using contact preheaters. In addition, high preheat temperatures result in the higher surface roughness of a product [95].

Kerbiriou and Friedrich [144] experimentally studied basic manufacturing parameters, namely temperature conditions on the preheater, heated die and cooling die, pressure in the heated die, and pulling speed, and their influence on density and mechanical properties. At the same time, Bechtold et al. [145] studied the effects of preheating, heated die temperature, and pulling speed on the mechanical characteristics by using glass fiber–polyamide 6 (Nylon 6) microbraided yarn. The influence of preheating temperature on the properties of produced profiles was determined by Evstatiev et al. [146].

### 2.2. Temperature and Geometry of the Heated Die 

**Figure 4:**
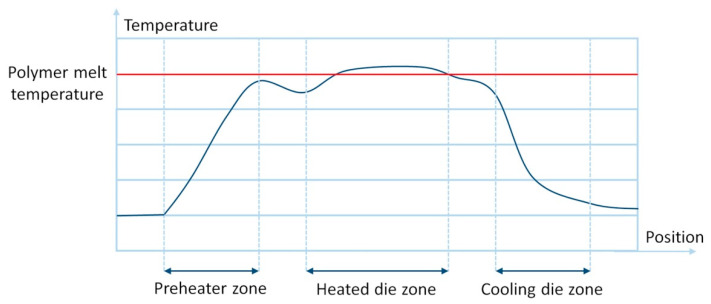
Typical distribution of temperatures inside the pultrusion machine.

The main component of the nonreactive pultrusion machine is the heated die block. The purpose of the die block is to melt the matrix, to impregnate fibers, and to impart a shape to the composite. To control the process of pultrusion, manufacturers equip the heated die block with thermocouples, pressure gauges, and electrical heaters [147]. On the one hand, the increase in temperature lowers the viscosity of the matrix, and increases pressure due to the thermal expansion, thus improving the impregnation of fibers [98]. As shown experimentally by Carlsson and Astrom [95], the increase in temperature results in better mechanical performance of the glass fiber and polypropylene (GF/PP)-based composite. On the other hand, the maximum temperature is limited by the temperature of thermal degradation of polymers [148], which, if exceeded, can result in polymer burn-out and rejected products. High pressure and temperature may cause the fracture of reinforcing fibers. Also, low viscosity in combination with low pulling speed and high pressure may force the matrix to move backward and accumulate at the entrance of the heated die [98]. Figure 4 shows the typical distribution of temperatures during the pultrusion process [149].

Experimental trials with different die geometries were carried out by Michaeli and Jurss [147]. Evstatiev et al. explored the influence of heated die temperature on the properties of pultruded profiles, using scanning electron microscopy, wide-angle X-ray scattering, and mechanical testing [146]. In the study with jute fiber composites [108], Memon et al. observed an increase in flexural strength at a certain temperature; however, further increase in temperature resulted in a reduction thereof. Schafer and Gries [150] proposed the unconventional heating method for braided pultrusion process. Simultaneously, Oswald et al. [151] analyzed the influence of temperature regime on the void content of thermoplastic pultruded profiles based on natural fibers, and investigated the influence of heating conditions on the void content. Optimized parameters of the pultrusion process (temperature conditions in particular) were investigated by Wongsriraksa and Nakai [152]. In [153], the effects of heating conditions on the mechanical performance of carbon fiber reinforced polymer (CFRP) composites were experimentally evaluated. Chen et al. [154] analyzed correlation between die temperature and properties (crystallinity, melting point, mechanical properties) of the manufactured profiles. At the same time, Lapointe and Laberge Lebel [149] investigated the use of a multi-die system for the better impregnation of thermoplastic pultruded rods.

**Figure 5:**
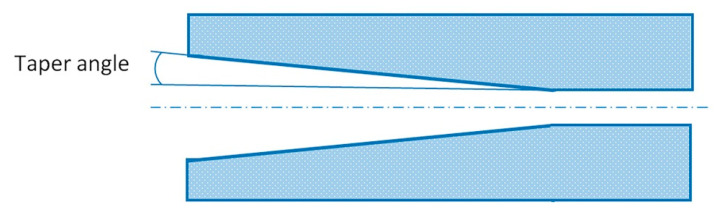
Angle of taper of the heated die.

Another important parameter affecting the impregnation of fibers is the geometry of the heated die block [149], the inner part of which has a tapered section linearly narrowing to the die exit (Figure 5) [155,156]. Near the exit of the die block, the cross-section becomes constant and assumes the geometry corresponding to the desired shape of a composite [157]. The tapered section of the die block is described by the angle of taper that affects the pressure and backward motion of the thermoplastic melt. In order to minimize friction between a composite and internal surfaces of the die block, and to reduce the pulling force, the internal surfaces of the die block are chromium plated [158]. In addition to the tapered die block designs where the reinforcement pack is shaped and impregnated by way of pressure exerted upon a material by internal surfaces of the die block, there is also a die block design where the thermoplastic melt is forced into fibers by special pins [97].

### 2.3. Heated Die Pressure

The process of fiber impregnation depends on the pressure. Pressure, in turn, depends on the viscosity of a polymer, pulling speed, and the angle of taper [157]. Pressure in a die block originates from thermal expansion of a polymer inside a tapered die block [147]. It is very difficult to evaluate the influence of pressure on the quality of a composite experimentally, as high pressure values can only be achieved at high pulling speed that adversely affects the quality of material because of resulting high void content [147]. Fanucci et al. [159] manufactured special sensors and studied their application for pressure registration during thermoplastic pultrusion.

### 2.4. Temperature of a Cooling Die 

The profile exiting the die block can lose its shape under external forces due to plasticity of the polymer at high temperatures. To prevent the loss of shape, it is necessary to cool the profile below the glass transition temperature [147]. As the profile already has the desired shape at the cooling stage, the cooling die has a constant cross-section. In order to achieve a sharp temperature gradient, the distance between the heated and cooling dies is rather small [157]. The experiments by Carlsson et al. [95] and by Kerbiriou et al. [144] show that cooling temperature influences the surface roughness of a product, and its flexural and shear strength.

Astrom et al. [143] experimentally investigated the influence of process parameters in general, and of the cooling die in particular, on the degree of crystallinity, and, therefore, on the mechanical properties of thermoplastic composites. More recently, Michaeli and Blaurock [160] discussed the relationship between cooling zone parameters and surface quality of produced profiles. Ghaedsharaf et al. [161] studied the effects of cooling die temperature and pulling speed on the resin impregnation, void content, and quality of the final surface.

### 2.5. Pulling Speed

The most important pultrusion parameter affecting all other parameters is the pulling speed. The pulling speed determines the time the reinforcement and a polymer stay within the preheater and inside a die block. Impregnation, pressure within the heated die block, pulling force, heating uniformity, and viscosity of thermoplastic melt—all depend on the pulling speed [98]. It was experimentally established that reduction in flexural strength is associated with increase in a pulling speed [162,163]. In addition, the increase in a pulling speed can adversely affect the shear strength and interlaminar shear strength [162]. Carlsson and Astrom [95] observed formation of defects at specimen surfaces with the increase in pulling speed. They attributed it to matrix sticking to the internal surfaces of the heated die block due to high pulling speed and high cooling temperature. Wiedmer and Manolesos also observed the shift from glossy to rough surfaces [98].

The relationships between pulling speed and compressive, flexural, and interlaminar shear strength of thermoplastic pultruded composites were experimentally analyzed by Astroem et al. [164]. Cho et al. [139] investigated the influence of pulling speed, heating temperature, and the reinforcement volume fraction on the temperature evolution of the resin, its conversion, and physical and mechanical characteristics. At the same time, aiming to achieve higher pulling speeds, Squires et al. conducted an experimental study by varying heating and cooling temperatures, as well as pressure profiles [165]. Azari [166] investigated the influence of pulling speed on the wet-out and mechanical properties of pultruded strands. Seeking to optimize pulling speed, Ozturk et al. explored the sensitivity of the process to changes in the pulling speed by changing the manufacturing parameters of the pultrusion line [167]. Subsequently, the effects of pulling speed on the microstructural and mechanical characteristics of pultruded profiles were investigated by Evstatiev et al. [146]. Nunes et al. [168] analyzed the influence of pulling speed and heating temperature on the mechanical and physical properties of pultruded profiles manufactured of towpregs. The effect of pulling speed on the mechanical performance of CFRP composites was evaluated by Wongsriraksa and Nakai [153]. Pulling speed optimization in the case of glass-fiber-reinforced polyamide-6 (PA-6) composite manufactured by thermoplastic reaction injection pultrusion technique is discussed in [154]. Simultaneously, Lapointe and Laberge Lebel investigated effects of pulling speed on the void content and quality of impregnation [149].

### 2.6. Pulling Force

Pulling force can change depending on the pulling speed, section geometry, taper angle, and viscosity of a polymer. The critical value of pulling speed should be tightly controlled in order to prevent production interruptions and to maintain the integrity of a profile [169]. As shown by Carlsson and Astrom [95], the pulling speed is the main factor affecting the pulling force. Astrom [155] succeeded in establishing the relation between the taper angle and pulling force. At angles exceeding 5°, the pulling force is relatively low; the drastic increase in pulling force is observed with the decrease in the angle of taper. In addition, the increase in the perimeter of a profile cross-section also results in the increased pulling force. The correlation between pulling force and pulling speed was experimentally investigated by Nakai and Morino [170].

### 2.7. Future Trends 

Analysis of the thermoplastic pultrusion process and its parameters, as well as consideration of thermosetting pultrusion scientific and industrial state-of-the-art, demonstrate that deeper research is needed to better understand the peculiarities of the thermoplastic pultrusion process. The deeper knowledge of the thermoplastic pultrusion process will stimulate the interest in this manufacturing technique from the scientific and engineering community. This subchapter briefly discusses promising directions for future investigations in this field. All the topics listed below require careful research, since there are currently few publications available on the mentioned subject, or research has not been conducted at all.

Although some relationships between process parameters and mechanical characteristics of the thermoplastic pultruded products have been established, extensive experimental research is needed to understand the direct influence of these parameters on each of the following mechanical properties at different loading conditions and strain rates: tension, compression, flexure, buckling, shear, creep, and fatigue. Degree of crystallinity, melting, and consolidation evolution during polymerization depends on the temperatures used; thus, a deeper understanding of this interconnection is necessary in order to improve process outcomes. The influence of the die geometry defining the thickness of the manufactured profiles and, thus, determining mechanical properties and shape distortions of the final product, also requires detailed research. Moreover, severity of process-induced shape distortions both in thermoset [12,23,171] and in thermoplastic pultrusion depends on the temperature and, therefore, is another potential field of investigation. The relationship between process parameters and formation of voids, cracks, and delaminations also require in-depth research. A successful application of thermoplastic pultruded structures in harsh and severe environments will require better understanding of the influence of process parameters on the service life of the produced profiles.

Finally, in order to avoid expensive trial–error experiments when studying the influence of process parameters of the thermoplastic pultrusion, we need better optimization and numerical simulation algorithms. Moreover, the existing models require refinement to improve control over process parameters and to obtain better outcomes of the thermoplastic pultrusion process.

The studies discussed above mostly deal with profiles of simple cross-sections (rods, flat profiles). Currently, there is a lack of studies describing the thermoplastic pultrusion of complex shape profiles, such as pipes, channels, I-beams, decks, etc., commonly used in the construction industry. Despite the large number of publications on pultrusion with unidirectional reinforcement, there are no studies on thermoplastic pultrusion with various reinforcement types, and on application of fabrics, mats, and veils. Moreover, there are no studies on the stability of the thermoplastic pultrusion process; i.e., how many profiles (particularly of complex shape) of steady, acceptable quality can be produced within a single manufacturing cycle. Furthermore, successful scaling, development, and industrial application of thermoplastic pultrusion will require more studies on manufacturing process controls and elimination of defects.

## 3. Raw Materials and Properties of Obtained Composites 

Final properties of a material depend both on the quality of manufacturing and on the quality of raw materials. The main problem in thermoplastic composite manufacturing is the need to ensure good impregnation of reinforcing fibers with matrix, as the viscosity of thermoplastic polymers is significantly higher than that of the thermosetting ones, e.g., the average viscosity of thermosetting polymers is 0.03–1 Pa∙s [142], as opposed to 500–5000 Pa∙s [172] in case of thermoplastic ones. One way to simplify the process of nonreactive thermoplastic pultrusion is the use of prepregs where reinforcing fibers are in the close contact with matrix uniformly distributed over the whole length of a prepreg. When the prepreg enters the die block, thermoplastic polymer contained in the prepreg will melt and impregnate the fibers under pressure. Table 1 and Table 2 show properties of some polymers and fibers used in the thermoplastic pultrusion.

Longmuir and Wilcox proposed a novel technique allowing a variable number of fiber strands to be used during the manufacturing process [173]. Thomasset et al. performed a rheological study on the polypropylene and long-glass-fiber composites manufactured by pultrusion [174]. Simultaneously, Broyles et al. [175,176,177] studied the influence of fiber sizing agents on the mechanical properties and moisture absorption. Next, Roy et al. [178,179] succeeded in improving compression behavior of pultruded composites by modifying material composition and parameters of the thermoplastic pultrusion process. Subsequently, Fink and Ganster [180] conducted an experimental study of the influence of synthetic fibers and of the choice of matrix on mechanical properties of composites. A novel tool intended for the manufacturing of thermoplastic pultruded profiles was proposed by Novo et al. [181]. Tao et al. [182] analyzed mechanical performance, thermal stability, and morphology of composites based on long- and short-glass-fiber reinforcements. The influence of fiber content on mechanical and tribological properties, morphology, and thermal stability of pultruded polyoxymethylene (POM)–basalt fiber composites was studied by Wang et al. [183]. Kahl et al. [184] used different types of reinforcement (cellulose and glass fibers) and matrix material (polypropylene and polyamide) to evaluate the influence of raw materials on the mechanical performance of manufactured specimens. Shayan Asenjan et al. [185] conducted the experimental study to understand a correlation between the length of fibers and high-velocity impact performance. Chen et al. [154] investigated the influence of volume fraction of reinforcement on the density, heat distortion temperature, void occurrence, and mechanical characteristics of the glass fiber–polyamide-6 (PA-6) composites produced by RIM pultrusion. Recently, the relationship between impregnation and mechanical properties was studied by Saito et al. [186]. At the same time, seeking for a reduction in carbon footprint, Asensio et al. [187] studied the possibility to use recycled material for the pultrusion of thermoplastic composites.

Various additives (fillers), with the most popular being nanotubes, can improve the performance of composites. Nanotubes improve interlaminar shear strength, interfacial shear strength, and delamination resistance of a composite [201,202]. Addition of Ni powders increases the flexural modulus; the optimal ratio of matrix, filler, and Ni powder improves the mechanical performance of composites [81]. Various fiber coatings make it possible to improve tensile, compression, and flexural strength of a composite with a 2% increase in material weight cost [175]. Markov [203] showed how the distribution of filler particles within the pultruded composites affects their electric characteristics. Recently, Chen et al. [141] experimentally analyzed the influence of activators and initiators on the polymerization process.

**Figure 6:**
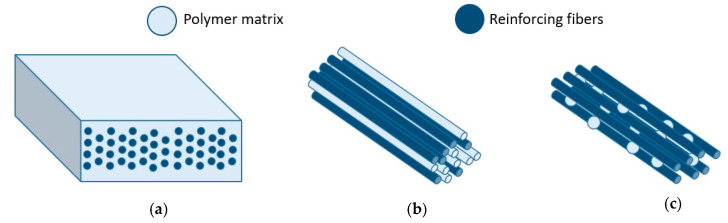
Prepregs schemes: (**a**) preconsolidated tapes, (**b**) commingled yarns, and (**c**) towpregs.

Several prepreg types for thermoplastic pultrusion are currently available on the market: preconsolidated tapes (Figure 6a), commingled yarns (Figure 6b), and towpregs (Figure 6c) [192,204]. Seeking to optimize thermoplastic pultrusion process, Iftekhar [205] explored the influence of fillers and additives on the viscosity of resins. The relationship between width/thickness of the prepregs and the mechanical and physical properties of composites was studied by Mariatti [206]. Hedayati Velis et al. analyzed the influence of polymer matrix and of a series of prepregs on the mechanical properties of pultruded composites [198].

### 3.1. Preconsolidated Tape 

**Figure 7:**
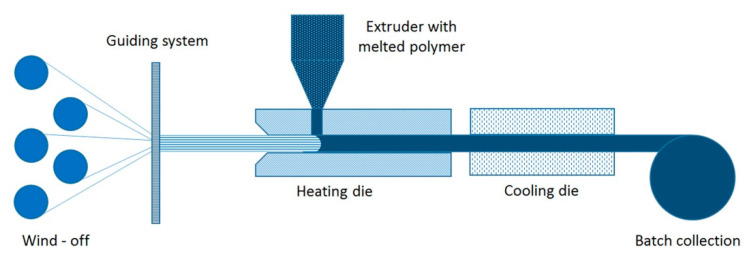
Schematic illustration of a preconsolidated tape (PCT) production machine.

Preconsolidated tape (PCT) consists of reinforcement fibers impregnated with a thermoplastic polymer at a specific volume fraction. The PCT fabrication method is similar to that of pultrusion—hot thermoplastic melt is injected into the heated die block [192]. The material is then cooled and wound onto reels for storage and transport. Figure 7 shows the schematic illustration of a PCT production machine [192].

Currently, produced PCT can have widths of up to 300 mm and thicknesses of 0.125–0.500 mm [188]. The most popular PCTs are produced with fiber volume fraction of 60%, at the rate of 20–60 m/min. PCT can be produced in a towpreg production line with the additional heated die installed at the end of the line [194,207].

### 3.2. Commingled Yarns (CY)

**Figure 8:**
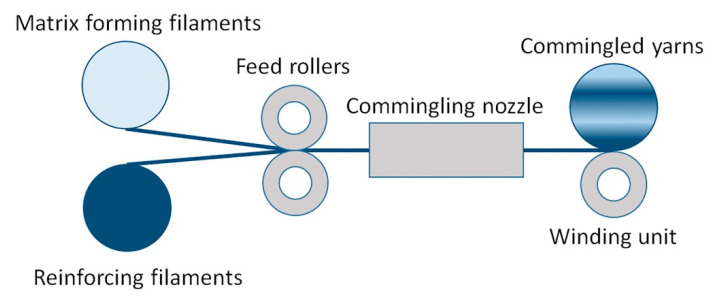
Commingled yarn fabrication.

Commingled yarns (CY) are composed of intermingled matrix and reinforcement filaments [208]. One of the ways to manufacture CY is a mixing of fibers during winding with the use of a winding machine (Figure 8) [188]. In CY production it is possible to ensure uniform distribution of matrix and reinforcement filaments over the whole length of prepreg while maintaining the desired volume fraction of reinforcing material [99]. Distribution of filaments plays a very important role, as it affects flexural performance of a composite, its specific weight, and fiber volume fraction in a composite. There are four types of mixed fibers prepregs currently available on the market: commingled, cowrapped, core-spun, and stretch-broken yarns [172]. The most popular are commingled yarns [209,210,211,212,213]. Under pressure from thermoplastic melt, reinforcing fibers tend to aggregate during the impregnation and form agglomerations (Figure 10) [213].

### 3.3. Towpregs

**Figure 9:**
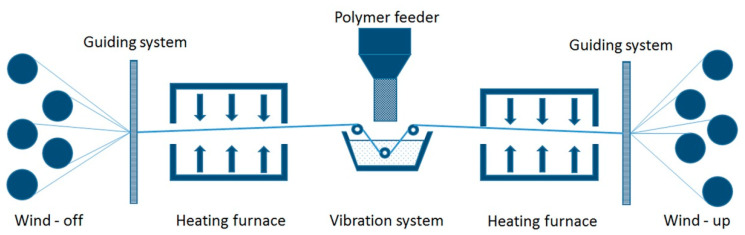
Schematic illustration of a towpreg production machine.

Towpregs fabrication consists in mixing fine-powdered thermoplastic polymer with reinforcing fibers. In dry methods of towpregs fabrication, reinforcing fibers pass through the chamber with powdered polymer and are further fed into the heated chamber where thermoplastic polymer is ultimately joined with reinforcement fibers. In 2000, a pultrusion head for producing towpregs materials was patented at the University of Minho [214]. By convention, towpreg machines consist of five components: fiber creel, guiding system, powder feeder, heating chamber, and winding mechanism (Figure 9) [192]. The powder feeder can utilize various powder agitation systems, such as pneumatic [194], vibration [192], and electrostatic [188]. To handle the problem of high viscosity of thermoplastic melts, the alternative method of wet fabrication can be applied, where a solution of thermoplastic polymer in a solvent is used for impregnation. Reinforcement fibers are impregnated with a solution of thermoplastic polymer and then placed into a heated chamber to evaporate solvent, leaving the neat thermoplastic polymer on fibers. However, as the solvent is quite difficult to remove completely, this can result in increased porosity of the finished composite [188]. Optimization of the towpregs manufacturing process by means of Taguchi’s DOE (design of experiments) method was performed by Novo et al. [215]. Nunes et al. [168] studied the influence of pulling speed and furnace temperature on the polymer content in towpregs.

### 3.4. Mechanical Properties of Obtained Composites

Typically, the same standards are used in mechanical testing of thermoplastic and thermoset pultrusion samples. For instance, ASTM D6641-16 is used for compression [216], ISO 527-5 is used for tension [217], ASTM D790-15e2 is used for flexure [218], ASTM D7078/7078M-12 is used for in-plane shear [219], and ASTM D2344-16 is used for interlaminar shear testing [220]. For comparison purposes, we have listed some mechanical properties of pultruded thermoplastic (Table 3) and thermoset (Table 4) [1,30] matrix composites.

### 3.5. Durability of Thermoplastic Pultruded Materials 

If we want pultruded thermoplastic profiles to be widely used, then we need be sure of their durability. Unfortunately, durability of both thermoset [223] and thermoplastic pultrusion has not been studied deeply enough. Articles published are mostly related to topics other than pultrusion technology. We intend to analyze reaction injection molding and press molding in brief in order to attract scholars’ and engineers’ attention to thermoplastic pultrusion durability.

Under microorganism actions, the molecular structure of a polymer can biodegrade and both physical and chemical properties can also change. Polymers can be the source of energy for the microorganisms [224]. The molecular bonds can be destroyed, as well as the composite’s properties. Biodegradation depends on crystallinity, temperature, pH of the environment, humidity, molecular weight of the polymer, various additives with enzymes, and bioorganisms [225]. Not all plastics are biodegradable, only some, such as polyvinyl alcohol (PVA), polycaprolactone (PCL), polyester, polylactide (PLA), polyethylene, nylon, polyhydroxybutyrate (PHB), and polyglycolide (PGA) [226,227]. Some composites are nonsusceptible to the process of biodegradation; thus, starch polymers [228,229,230,231,232,233,234,235] and fish waste [236] additives are used to accelerate the process. Composite materials based on natural fibers can be of great interest as they fully recycle through biodegradation. Moreover, biocomposites can be recycled in composting conditions [224,233,237]. Reinforcements based on wood [238], aspen [239], flax, hemp, sisal [240], cellulose [241], pineapple leaves [242], and reed [243] are typically applied. Degradation rate depends on the structure of the natural fibers, like the flexural strength of composites based on nonwoven mat decreases more than that of a woven composite [237]. 

Although the reaction of polymerization in thermoplastics composites is complete, the shelf life of the polymers is virtually unlimited [51,52,53,54,55,56,57]. The degradation of material properties may occur over time due to fatigue loads, temperature exposure, humidity, chemical reactions, radiation, etc. For example, fatigue provokes fiber failure, matrix cracking, interface debonding [244], and decrease in material strength [245]. 

Polymers behave differently depending on heating conditions and temperatures. Fatigue strength decreases faster at cryogenic temperature comparing with room temperature [246]. Long-term aging of composite material at a temperature below melting causes a change in glass transition temperature and strength [247,248]. During short-term aging, thermoplastic composite is sharply heated to thermal decomposition temperature; thus, rapid decrease in tensile and interlaminar shear strength is observed [249,250]. Apart from debonding and cracks, delamination and fiber failures can occur [251,252]. Freeze and thaw cycles, accompanied by cooling the material below 0 °C, lead to loss of flexural strength and Young’s modulus [253].

Water immersion and exposure to humidity affects tensile, compression, and flexural strength [254]. Mechanical properties depend on the temperature of the surrounding medium [254]. Typically, thermoplastic composites are studied in seawater [254,255,256], tap water [257,258], etc. Acid and harsh environments negatively affect the molecular bonds and mechanical characteristics of the polymers [259]. Various gases, such as air, air under pressure, and nitrogen, reduce the strength of composites [260]. 

Research on thermoplastic pultruded material behavior, when placed in a different harsh environment, is needed if we want wide use of profiles in marine and chemical engineering. Nuclear engineering is interested in the study of reactive radiation effects. Durability of both matrix and fiber reinforcement is to be studied as well. Experiments conducted at high and low temperatures are needed to apply the thermoplastic pultruded profiles for civil engineering in different climatic zones.

### 3.6. Future Trends 

The properties of the pultruded profiles depend greatly on the choice of raw materials. The lack of knowledge in the field of thermoplastic pultrusion becomes apparent in the choice of raw materials, as opposed to the thermoset pultrusion. To popularize the application of thermoplastic pultrusion, we need better understanding of physical phenomena taking place during the manufacturing process, and their dependence on the choice of raw materials, and clearer understanding of the influence raw materials have on mechanical performance and the life cycle of thermoplastic composite materials and structures. This subchapter will briefly discuss the most promising areas of research, from the authors’ point of view. 

In spite of availability of several studies on the influence of raw materials on mechanical performance of pultruded thermoplastic composites and structures, we believe that more research is necessary in order to study this question. Of special importance here are the studies of stress–strain state in composite materials under different modes and rates of loading. 

An in-depth research of existing and perspective additives is necessary to better understand their influence on the properties of end products and to improve mechanical performance and physical properties of pultruded thermoplastic profiles. Currently, only the studies by Markov [203] and Chen et al. [141] are available in this field. In addition, there is an obvious lack of studies on the influence of micro- and nanoadditives, both in thermoset pultrusion (Kuruvilla and Renukappa [261], Manjunath et al. [262]) and in the thermoplastic one (Roy et al. [263], Alam et al. [264]).

Striving to reduce environmental footprint, human society demonstrates ever increasing interest in sustainable development and the use of natural materials, and the composite industry is no exception. Application of biocomposites is currently one of urgent research topics in thermoplastic materials [265,266,267,268,269,270]. Broad introduction of such composites into everyday life will obviously require extensive study of their properties and characteristics.

While the influence of additives improving UV-aging performance and corrosion resistance of end products is mostly well understood for the thermoset pultrusions, this is not the case for the thermoplastic ones. Therefore, the real application of thermoplastic composites will require extensive studies of their behavior in the presence of various additives, which might be of special interest for the industrial and scientific community. In addition, of great interest for the composite community is the influence of nonbiodegradable and flame-retardant compounds on the properties of thermoplastic materials.

The use of hybrid reinforcements (e.g., the simultaneous use of glass and carbon reinforcing fibers) is currently one of the hottest topics, since some loaded parts of pultruded structures may benefit from the use of different fiber types. However, there is a lack of knowledge on this issue both in thermoset [271] and, particularly, in thermoplastic pultrusion.

## 4. Process Modeling

Manufacturing of pultruded thermoplastic composites depends on various process parameters such as preheater temperature, heated die geometry, temperature and pressure inside a heated die, cooling die temperature, pulling speed, and pulling force, and, thus, necessitates the development of mathematical models for process optimization. In addition to process parameters, the properties of prepregs, such as melting temperature, glass transition temperature, coefficient of thermal expansion, etc., should also be taken into account. All things considered, mathematical models should allow an engineer to determine the degree of impregnation, temperature distribution, pulling force, etc., for complex profile geometries. These models, based on modern methods, would allow calculation of residual stresses in a composite, making it possible to predict cracking, warping, shrinkage, and other process-induced deformations. Among the published articles and books on mathematical modeling in thermoplastic pultrusion, of particular interest is the book by Suresh Advani and Murat Sozer [272]. Currently, several studies of residual stresses and strain are underway in the field of thermoset pultrusion [30,171,273,274,275], while the number of similar studies for thermoplastic pultrusion is significantly lower. An overview of existing mathematical models of nonreactive thermoplastic pultrusion follows.

### 4.1. Impregnation

**Figure 10:**
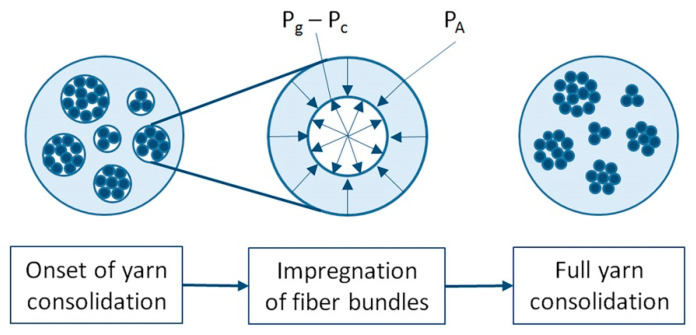
Schematic illustration of yarn section and consolidation process; Pg—pressure from the void, Pc—capillary pressure, P_A_—applied pressure.

Properties of final products depend on the fiber volume fraction. The volume fraction and strength of material, in turn, depend on fiber impregnation [276] impeded by the high viscosity of thermoplastics. Several mathematical models were developed to find optimum manufacturing conditions and to investigate the relationship between the degree of impregnation and process parameters. Most of these models describe the nonreactive thermoplastic pultrusion with commingled yarns and are based on the following approximations:

- Reinforcing fibers are represented by separate groups (agglomerations) in the thermoplastic melt (Figure 10);
- These groups have an elliptical or circular section;
- Fibers are impregnated uniformly over the bulk of the product on all sides.

The aim of these models is to determine the degree of impregnation at any moment of time and to estimate the void content [209,213]. The motion of thermoplastic melt through fibers is governed by Darcy law describing the flow of fluid through a porous medium [277]:

(1) $$u=-\frac{K}{\mu }\nabla p,$$

where u—the speed of fluid motion inside fibers, K—fiber permeability tensor, µ—viscosity, ∇—nabla operator, and p—external pressure.

Taking into account the fiber volume fraction and local speed of fibers and thermoplastic melt, Bernet et al. [213] obtained the following expression for Darcy law:

(2) $$\left(1-V_{f}\right)\left(u_{l}-u_{s}\right)=\;-\frac{K}{\mu }\nabla p,$$

where $V_{f}$—fiber volume fraction, $u_{l}$ and $u_{s}$—local speeds of thermoplastic melt and fiber, respectively.

As the permeability K is not constant and uniform in all directions, the impregnation process is difficult to model. In order to calculate permeability in the direction parallel to fiber orientation, the Kozeny–Carman equation is used [211]:

(3) $$K_{∥}=\frac{r_{f}^{2}\cdot {\left(1-V_{f}\right)}^{3}}{4\cdot k_{0}\cdot V_{f}^{2}},$$

where $r_{f}$—fiber radius, $V_{f}$—instantaneous fiber volume fraction depending on the pressure, and $k_{0}$—the permeability constant.

To determine permeability of fibers in transverse direction, the equation proposed by Gutowski et al. is used [278]:

(4) $$K_{⊥}=\;\frac{r_{f}^{2}{\left(\sqrt{\frac{V_{a}}{V_{f}}-1}\right)}^{3}}{4k_{0}\left(\frac{V_{a}}{V_{f}}+1\right)},$$

where $V_{a}$—maximum possible fiber volume fraction and $k_{0}$—the permeability constant.

When modeling the impregnation process during pultrusion, one should consider the motion of matrix along the fibers. Kim et al. [212] proposed the micromodel describing the impregnation of fibers in a transverse direction, and the macromodel describing the lengthwise flow of matrix. The macromodel was based on Darcy law and the Kozeny–Carman equation. Later, the model was supplemented with mass conservation equations in a cylindrical coordinate system (Equation (5) for matrix, and Equation (6) for fiber reinforcement):

(5) $$\frac{\partial }{\partial t}\left(1-V_{f}\right)+\frac{1}{r}\frac{\partial }{\partial r}\left(\left(1-V_{f}\right)ru_{l}\right)=0,$$

(6) $$\frac{\partial }{\partial t}V_{f}+\frac{1}{r}\frac{\partial }{\partial r}\left(V_{f}ru_{s}\right)=0,$$

where r—matrix propagation front.

Koubaa et al. [279,280] have developed a pultrusion impregnation model based on the Navier–Stokes equation, having the following expression in a cylindrical coordinate system:

(7) $$\frac{\partial P}{\partial z}=\frac{\mu }{r}\frac{\partial }{\partial r}\left(r\frac{\partial u_{z}}{\partial r}\right),$$

where  z—coordinate axis coinciding with the pulling direction and $u_{z}$—fluid motion speed along the longitudinal axis. The following boundary conditions are imposed: zero component of fluid motion speed outside fibers, and equality of fluid motion speed inside fibers and of the pulling speed.

The model proposed by Gibson et al. [281] takes into account the capillary force. Sala and Cutolo [163] conducted numerical and experimental studies and proposed the model that uses both Newtonian and power-law relationships to predict the impregnation process outcomes. At the same time, Haffner et al. [282] developed a mathematical model that describes the microscopic flow of resin and accounts for different fiber arrangements, volume fraction of reinforcement, and impregnation time. Miller et al. [38] proposed the impregnation model for a composite material based on towpregs. The model represents a cell consisting of three filaments with two thermoplastic particles incorporated in spaces between filaments. Melting thermoplastic particles impregnate the filaments and fill the space between them. The proposed model considers the external pressure exerted by the fiber bed, capillary pressure, viscous pressure resulting from matrix motion, and springing pressure from fiber compaction. The resulting equation for impregnation time accounts for filament diameter and the size of particles, assuming them constant over the whole bulk of material. Results obtained with the analytical model are close to the experimental data, demonstrating good accuracy of the model. Subsequently, Bechtold et al. [283] proposed two different methods to model the impregnation process in the case of thermoplastic pultrusion with braided commingled yarns. Koubaa et al. [284] studied the impregnation of a single glass-fiber bundle and proposed the model based on the Young–Laplace law that takes into account the influence of capillary force. Ngo et al. [285] proposed a model of thermoplastic pultrusion with carbon fiber-reinforced prepreg that accounts for a multiscale 3D impregnation die. 

### 4.2. Temperature Distribution

The thermal model makes it possible to determine the distribution of temperatures over the heated die and the degree of crystallinity of a thermoplastic polymer. All developed models rely on the heat transfer equation adapted to the pultrusion process, which results in introduction of a pulling speed term on the left side of the following equation [286]:

(8) $$\rho \;CV\frac{\partial T}{\partial x}=\frac{\partial }{z}\left(k\frac{\partial T}{\partial z}\right)+Q,$$

where ρ—specific density, C—specific heat capacity, V—pulling speed, *x*—coordinate axis parallel to the die block axis, *z*—coordinate axis perpendicular to the die block axis, T—temperature, and Q—energy released during crystallization. Most polymers are amorphous and do not form a crystal lattice [286], making it possible to disregard the *Q* variable in most cases.

Using the model proposed by Åstroöm and Pipes [169,287], Babeau [288] conducted the experimental test and numerical simulation, and obtained results that are very close to the analytical model. Carlsson [96] proposed the following expression for the energy released during crystallization of polypropylene:

(9) $$Q=m\frac{\partial \alpha }{\partial t}H,$$

where m—mass fraction of a polymer matrix, α—degree of crystallinity, and H—theoretical ultimate heat of crystallization at 100% crystallinity.

The crystallization process can be divided into two stages. The first stage is the formation of primary nuclei. The second stage is the growth of a crystal formed on the nuclei. Thus, the derivative of the degree of crystallinity can be expressed as follows [96]:

(10) $$\frac{\partial \alpha }{\partial t}\left(T,\;\alpha \right)=\left(f_{1}\left(T\right)+f_{2}\left(T\right)\alpha \right)\left(1-\alpha \right),$$

where the $f_{1}$ function accounts for formation of primary nuclei and $f_{2}$ accounts for further growth of the crystal. Expressions for $f_{1}$ and $f_{2}$ were proposed by Malkin [289]. In addition to analytical methods, scientists often use numerical methods to determine the degree of crystallinity and the heat transfer, as explained by Haffner et al. [290]. Trying to minimize modeling oscillations in the case of high pulling speed and rough mesh, Ruan et al. [291] developed a 2D thermal model of thermoplastic pultrusion. Subsequently, Yn et al. [142] utilized the finite difference method to predict temperature and reaction evolutions within the pultruded profile and to optimize process parameters while maximizing the thickness of pultruded profile. Nejhad [148] proposed and verified experimentally a numerical model dealing with thermal analysis of impregnated tows/tapes in thermoplastic pultrusion. Numerical modeling providing information on both temperature distribution within a profile during pultrusion and crystallization kinetics of the polymer was proposed by Carlsson and Astrom [292]. Ahmed et al. [293] applied the FE–NCV (finite element–nodal volume control) approach to determine the heat distribution over the heated die block and to calculate the degree of crystallinity. Aside from Ahmed, Joshi and Lam [294] used the same modeling approach to investigate crystallization in a composite based on carbon fibers and PEEK polymer (CF/PEEK) specimen. 

### 4.3. Pressure and Pulling Force

Åstroöm [287] proposed a model describing the distribution of pressure over the heated die and the model of pulling force. He used the integral relationship between the pulling force and the drag over the unit area, expressed in the following form:

(11) $$f_{tot}\left(x\right)=\left(1-\Omega \left(x\right)\right)f_{v}\left(x\right)+f_{c}\left(x\right)+\Omega \left(x\right)f_{f}\left(x\right),$$

where Ω(x)—the part of a composite subjected to pressure, $f_{v}\left(x\right)$—viscous drag for Carreau fluid, $f_{c}\left(x\right)$—compaction resistance resulting from fibers compaction in the tapered portion of the heated die block, and $f_{f}\left(x\right)$—friction resistance. 

The model [287] uses the Carreau model and considers the nonlinear nature of thermoplastic melt viscosity:

(12) $$\eta_{a}=\eta_{0}{\left(1+{\left(\lambda \gamma \right)}^{2}\right)}^{\frac{n-1}{2}},$$

where $\eta_{0}$—zero-shear rate viscosity, γ—shear rate, λ—indicates the shear rate at which shear-thinning effects become significant, and the dimensionless constant n describes the degree of shear thinning. The comparison of results obtained with the analytical model and experimental data can be found in [155]. 

Lee et al. [286,295] proposed a numerical model to predict pressure and pulling force, as well as temperature and crystallinity. Simultaneously, a model predicting pulling resistance from the die, together with temperature and pressure distribution within a composite was developed by Astrom and Pipes [155,296]. Parasnis et al. [297] studied the influence of viscosity and shear load on the pulling force. They used a finite element model and compared calculated pulling force values with experimental data. The authors reported a discrepancy between experimental and calculated data, appearing after a certain value of pulling speed was reached. While the model predicted an exponential increase in pulling force, the experiments demonstrated that the increase in pulling force takes place until a certain pulling speed value was reached; after reaching this value, the pulling force remained unchanged. Blaurock and Michaeli proposed a method predicting pulling force and then compared it with experimental data [298]. Stavrov and Tsvirko [179] analyzed the relationship between pulling force and viscous characteristics.

### 4.4. Future Trends

Existing impregnation, and temperature and pressure distribution models rely on a series of approximations allowing a determination of process parameters for simple profile and die shapes, and mostly for commingled yarns. However, modeling of thermoplastic pultrusion for complex cross-sections, towpregs, and PCT still remains an open issue. Thermoplastic pultrusion cannot boast significant progress in mathematical modeling as opposed to the thermoset pultrusion, where it widely applied, first, to model complex shaped profiles (L- and I-shaped sections [274], wind turbine blades [299,300], etc.) with complex reinforcement lay-ups, and, second, to develop algorithm for their optimization [301,302,303,304,305,306,307,308,309,310,311,312,313,314,315,316,317]. In recent years, a significant interest to optimization has been observed in the scientific and engineering community. Optimization tools allow engineers to solve a large number of problems, from pultrusion process optimization to optimization of raw materials for pultruded profiles, and to take full advantage of composite materials. However, in spite of certain advances in optimization, there is, still, a lack of knowledge and experience on multiobjective optimization, both in thermoplastic and thermoset pultrusion. Aside from process parameters optimization, multiobjective optimization makes it possible to optimize the geometric topology of composites [318]. Modern mathematical models should allow a solution of process optimization problems with large number of input parameters. Moreover, modeling methods may help researchers investigate residual stresses in a composite, and their influence on cracking, delamination, warpage, shrinkage, and other process-induced defects [171,275]. The authors believe that expanding the use of supercomputers will bring the problems of mathematical modeling and optimization of composites to a fundamentally new level. Thus, aside from solving problems discussed earlier, the growing computational power will allow us to explain and model macrobehavior of composite materials, based on their microscale parameters.

## 5. Application

### 5.1. Pultrusion Market

The pultrusion market demonstrates steady growth from year to year. According to the European Pultrusion Technology Association (EPTA) forecast [319], the pultrusion market is expected to reach the mark of €100 billion in 2022. This growth opens new opportunities both for the thermoset and thermoplastic pultrusion. Thermoplastic pultrusion steadily gains popularity along with the thermoset one, although at a slower pace. For the sake of comparison, the entire thermoplastic composites market, including, aside from pultrusion, all the other thermoplastics applications as well, is expected to grow from €22.2 billion in 2020 to €31.8 in 2025, according to the report titled “Thermoplastic Composites Market by Resin Type (Polypropylene, Polyamide, Polyetheretherketone, Hybrid), Fiber Type (Glass, Carbon, Mineral), Product Type (SFT, LFT, CFT, GMT), End-Use Industry, and Region-Global Forecast to 2025” [320]. The main factor limiting the application of thermoplastic pultrusions is the price, i.e., the availability of thermoplastic resins, since they cost more than those used in thermoset pultrusion. This is one of the factors restraining the growth in thermoplastics applications [320]. Therefore, lower production costs, and, thus, lower final price of the manufactured products could stimulate the demand for thermoplastic profiles, offering competition to thermoset profiles, although both reports should be considered in the context of coronavirus (SARS-CoV-2) COVID-19 pandemic situation. To illustrate, in November 2020, the Federation of Reinforced Plastics reported a 12.7% drop in production of glass-fiber-reinforced plastics in Europe, reaching the mark of 996,000 tons in 2020, which is the steepest drop since the global economic crisis of 2008–2009 [321]. According to the same report, pultrusion production volumes in 2020 plunged by 10.7%, making the pultrusion industry the least affected by the crisis, when compared to all other composite sectors. 

### 5.2. Patents

According to registered patents, one of the most common applications of thermoplastic pultrusion are thin [322,323,324,325], round, and rectangular profiles. Typically these cross-sections are utilized as wires [326,327] and their coatings [328], rods [329,330,331], pipes [332,333], and hollow profiles [334,335] used in the production of doors and windows [336,337], etc. Global IP Holding Co. LLC patented the method to produce parts of sandwich structures [338,339] using the pultrusion process. They also patented constructions that combine both metal elements and composite materials [340,341]. Various combinations of fiber structures are used; for example, some of the layers are made of unidirectional fibers [342], while the others are made with transversely oriented fibers [343], fabrics [344,345], or long-fiber thermoplastics (LFTs) [346]. Pultruded profiles are widely used in railway construction. A group of engineers from Pultrusion Technique Inc. developed a method for the production of rail clamps, providing the benefit of excellent corrosion resistance [347], as compared to their iron counterparts. In addition, they patented wavy profiles [348] and elements with asymmetrical shapes [349,350].

### 5.3. Current Applications of Thermoplastic Pultruded Profiles

Pultruded thermoplastic profiles effectively combine properties of thermoplastic composites with advantages of pultrusion as a manufacturing process. Offering improved toughness and fire resistance, thermoplastic composites find application in many industries. Pultruded thermoplastic composites have found wide application in aerospace and [67,69] aviation [64,66] engineering. For example, landing gear doors made of thermoplastic composites have lower weight, compared to their aluminum counterparts, are weldable, and can be recycled, as opposed to those made of thermoset composites [68,351]. Thermoplastic composites can be used to manufacture airplane flooring [68], ice protection panels protecting the fuselage [351], various interior elements [351], rivets for fastening [15,65], aircraft wings [68], radomes [68], and flaps [68]. Aside from aviation, thermoplastic composites are widely used in the automotive industry [59,60,62,63]. The thermoforming ability of thermoplastics allows fabrication of various complex shape parts, such as dashboard carriers [61], body structures [61], bumpers [58,61,352], wheel rims [353], and seat structures [61] (commonly produced from long-fiber thermoplastics (LFT) [354]), etc. In civil engineering [70,71], thermoplastic composites are used to manufacture airfoils for wind turbine blades [355], pipes [84,85], rebars [86,87] and rods [88,89,90,91], reinforcement for concrete structures [86], window profiles [83], elements of walls [72], flooring [72], exterior siding [72], and roofing systems [72,73]. In addition, composite poles used in powerline-supporting structures for energy grids are often produced by thermoplastic pultrusion [74]. Moreover, pultruded elements can be used in restoration of deteriorated structures and rehabilitation projects [75]. Aside from these applications, there is a demand from the marine [76,77,78,79,356] and oil/gas [80] sectors. In view of product recycling capabilities, thermoplastic pultrusion is the process of choice for production of semiproducts for LFT [357,358,359,360,361,362,363] and cork and pellet composites [364,365,366,367,368]. Pultrusion can produce prepregs for constant size LFT with specified length of fibers and precisely maintained fiber volume fraction.

In spite of a steady growth in application of thermoplastic pultrusions in auxiliary elements of structures in the last few years, there are no published articles, patents, or news on the application of such profiles in the design and construction of full-scale bearing structures, such bridges, cooling towers, etc. This can be explained by the lack of knowledge on the behavior of these types of structures, which are produced of thermoplastic pultruded elements. Scientists and engineers have yet to investigate the strength, buckling, creep, fatigue, and durability aspects of thermoplastic pultruded profiles as applied to the full-scale structures.

Advances and experience in implementation of such structures are almost completely lacking, as there are no relevant design codes. European [369,370,371] and US [372] design codes regulating the design of pultruded structures deal only with thermoset profiles, or have no clear mention of pultrusion type. Therefore, these design codes can only be freely applied in the design of thermoset pultruded structures, as they were specifically developed for these types of profiles. The peculiarities of thermoset and thermoplastic pultrusion processes will undoubtedly impose certain limitations on the behavior of profiles under a particular loading mode. The behavior of thermoplastic and thermoset profiles under the same load may differ significantly. Thus, to account for specifics of thermoplastic profiles, existing structural design codes should be revised or rebuild anew. This will require extensive experimental investigations, the results of which will be used as a basis for future design codes.

### 5.4. Future Trends

Advanced studies mentioned in this subchapter show that pultruded thermoplastic profiles can be applied both in traditional areas mentioned previously, and in some, at first sight, nonconventional ones. The authors believe it is important to draw the attention of the composites research community to these perspective fields of research.

Application of green technologies and materials in manufacturing is a hot topic both in the composite community and in other industries [373]. Multiple studies conducted in the last few years demonstrate that human society should do its best to thoughtfully use and recycle the products of its activity, and to minimize its carbon footprint. Recent studies in recycling of thermoplastic composites demonstrate the growing interest to this field. There are various mechanical, chemical, and thermal approaches to composites recycling. The main problem associated with the application of recycled composites lies in a degradation of mechanical properties of recycled fibers to be used in newly produced composites [374,375]. On the other hand, striving to minimize carbon footprint, researchers opened a new perspective on the application of polyethylene terephthalate (PET), typically used in worldwide packaging, as a raw material for thermoplastic pultrusion [187]. Thus, any investigations aimed at application of recycled and natural raw materials in thermoplastic pultrusion will have a good perspective.

The application of thermoplastic polymers in composites holds considerable promise for the use of welded joints; however, the performance of such joints is yet poorly understood and will require the analysis on a case-by-case basis. In spite of the large number of publications in a field of thermoplastics welding in general [376,377,378,379], there are no studies on the welding of pultruded thermoplastic composites in particular.

There are a few studies reporting on the medical application of thermoplastic pultruded profiles. Tanimoto et al. [380,381] manufactured and investigated the properties of pultruded glass-fiber-reinforced polycarbonate wiring for orthodontic applications. Engineers from Fraunhofer Institute for Production Technology developed the process allowing a pultrusion of thermoplastic elements as small as 1 mm in diameter, which can be used in medical applications [382,383]. Authors report the excellent compatibility with magnetic resonance imaging (MRI) techniques and good post formability. Thermoset pultrusion was used to fabricate guidewire appliances that were mechanically tested along with in vivo experiments on animals [384]. In addition, the appliance compatible with magnetic resonance (MR) was pultruded and tested in various MR-guided cases aimed to study the behavior of arteries [385]. Recently, an experimental study [386] demonstrated the feasibility of thermoset micropultrusion of 280 µm-diameter carbon fiber elements, and proposed the use of thermoplastic matrices as a recommendation for further research.

Invented in ETZ Zurich, continuous lattice fabrication (CLF) is a new additive manufacturing (AM) technique making it possible to print thermoplastic fiber reinforced polymers (FRPs) in three-dimensional space at any imaginary trajectory, with the help of robotic arms [387,388,389]. This approach combines both extrusion and pultrusion. Introduction of AM or robotic fabrication techniques into thermoplastic pultrusion manufacturing would definitely broaden the perspectives for thermoplastic pultrusion. Meanwhile, a novel technique proposed by the Institute of Plastics Processing at RWTH Aachen University presents hybrid pultruded profiles combining both a thermoset core and thermoplastic top layer, combining the advantages of both processes [390]. Combination of thermoplastic and thermoset pultrusions may certainly result in more efficient structural components, thereby exploiting the advantages of both manufacturing techniques.

As the human society demonstrates the increasing interest in colonization of Moon and Mars, there will be a large demand for space transport technologies and to produce various structures for space stations, power generation platforms [391], and other facilities necessary to settle on other planets. There are no considerable obstacles to shipping a pultrusion machine into space [3] to utilize the advantages of thermoplastic polymers [392,393]. British company Magna Parva, specializing in space research, plans to use pultrusion in space where no kind of production has been done before [394].

Typically, pultruded profiles are supposed to have straight shapes; however, pultrusion of nonlinear profiles can be accomplished as well. Curved pultruded profiles can be used to minimize the excessive deflection of structures, to implement components of complex shapes, or to impart individuality to architectural forms. Currently, fabrication of curved pultruded profiles is actively investigated in thermoset pultrusion [395,396,397]. However, similar studies in thermoplastic pultrusion are very limited [398], and, therefore, this issue requires further investigation.

According to the studies on urban planning and development, over 70% of world population will live in cities by 2025. Steadily growing migration from countryside to the cities forces telecom companies to search for solutions to today’s technology challenges. Large megapolises require effective and innovative information services and data transmission facilities. Offering data rates of 10–20 Gbps, 5G may be a solution to these problems [399]. Pultruded profiles, being transparent to radio frequency signals, are perfectly suited for use in the growing 5G network infrastructure around the globe [399].

In addition, the growing interest in smart polymers, materials able to change their physical and chemical properties under the influence of various external factors (pH, temperature, UV light, etc.) [400], may also apply to thermoplastic pultrusion. Of special interest are shape memory polymers, a subset of smart polymers, which are able to recover their shape under the influence of certain external factors [401,402]. These materials with their unique properties can have various applications —in medicine [403] and self-healing systems [404], and in aerospace [405], electronic [403] and civil [403,406] engineering.

## 6. Conclusions

This study reviews the state-of-the-art in thermoplastic pultrusion. We discussed the distinctive features of the process, materials used, patents registered, properties of pultruded profiles, industrial market situation, and applications of thermoplastic pultruded profiles. Application of thermoplastic polymers in pultrusion instead of thermoset ones makes it possible to improve the impact strength of structures, and offers the advantages of recycling, indefinitely long storage of source material, and application of welded joints of composite profiles. However, the limited number of studies in the field of pultruded thermoplastic composites makes it difficult to unveil the full potential of thermoplastic pultrusion. Trying to answer the question of the huge industrial, scientific, and experience gap existing between thermoset and thermoplastic pultrusion, we were able to develop recommendations on further research in the application of composite structures in general, and pultruded thermoplastic profiles in particular. We also recommend the research areas necessary to broaden the field of thermoplastic profiles application in order to obtain the knowledge sufficient for understanding the complex mechanics of thermoplastic composites, which is necessary to design complex critical structures currently built of thermoset profiles.

It must be noted that this review, being the first of its kind (as no review papers on thermoplastic pultrusion were published earlier), discusses only general questions and does not probe deeper into specific aspects of thermoplastic pultrusion and the materials produced. However, as there is an urgent need for such studies, in the near future we can expect publication of separate review papers concerning specific subtopics of thermoplastic pultrusion, such as, for instance, additives, structural design, durability of pultruded thermoplastic elements, process-induced shape distortions, biocompatibility, and natural materials, among others.

## Figures and Tables

**Table 1:** Polymers used as a matrix in thermoplastic pultrusion.

Material	Melting Point, °C	Glass Transition Temperature, °C	Density, g/cm^3^	Elastic Modulus, GPa	Tensile Strength, MPa	Flexural Modulus, GPa	Flexural Strength, MPa	Reference
PBT	230–223	31–60	1.21–1.38	1.8–2.5	40–55	1.9–2.8	76	[188,189,190,191]
PA 6	220	49–75	1.10–1.12	2.8	48–80	1.9–3.2	69–117	[189,191,192]
PA 66	268	60–70	1.06–1.12	2.8–3.9	30–85	1.2–3.7	86	[189,191,193,194]
PA 12	174–185	55	1.01–1.03	0.5–1.9	45–70	0.36–1.2	-	[191]
PP	160–175	−15–−8	0.89–0.92	1.0–2.0	28–41	0.8–1.7	45–55	[169,188,189,191,192,193,195]
PEEK	334–345	143–158	1.29–1.34	3.1–8.3	90–115	2.8–3.9	110	[149,169,188,191,192,196]
PEKK	360	154–171	1.27–1.31	4.0	110	-	-	[188,189,191]
PET	243–250	60–88	1.30–1.38	2.5–4.0	50–70	2.8	110	[188,189,191,193]
PEI	216–220	209–249	1.26–1.70	2.7–6.4	100–105	2.9–3.3	151	[188,189,191,196]
PES	220–238	220–246	1.36–1.58	2.4–8.6	83–126			[188,191]
PMAA	105–160	82–105	1.17–1.26	2.8–3.4	62	3.2	97	[191,193]
PPS	280–290	74–92	1.35–1.43	3.4–4.3	66–93	3.4–4.1	96–151	[188,189,191,192,196,197]
PLA	150–162	55–75	1.18–1.26	0.5–3.5	21–170	1.8–2.8	-	[189,191,195]
HDPE	130–137	−133–−118	0.95–0.97	0.7–1.4	20–40	1.2	-	[189,191,195,198]
LDPE	105–125	−133–−103	0.92–0.93	0.1–0.4	5–17	-	-	[189,191]
PC	255–267	−158–−134	1.18–1.22	2.4	55–75	2.1–2.4	80–93	[189,190,191,192,193]
PE	104–113	−133–−59	0.92	0.2	10–18	-	-	[188,191]
PU	220–230	−60–−19	1.15–1.25	0.1–0.7	5–28	-	-	[188]

**Table 2:** Fibers used as reinforcement in thermoplastic pultrusion.

Material	Density, g/cm^3^	Tensile Modulus, GPa	Tensile Strength, GPa	Poisson’s Ratio	Reference
E-Glass	2.5–2.54	70–73	1.5–2.3	0.20–0.30	[1,188,189,199]
S-Glass	2.46	90	4.5	0.21–0.23	[1,188]
Carbon	1.94–2.15	585–725	2.2–3.8	0.25–0.30	[1,188]
Flax fibers	1.5	50	0.5–0.9	-	[1,189]
Jute fibers	1.3	26.5	0.4–0.7	-	[200]
Hemp fibers	1.45	64	0.69	-	[189]
Graphite	1.90	3.3	-	0.28	[197]
Aramid	1.45	125	2.8–3.5	0.35	[188,199]

**Table 3:** Mechanical properties of thermoplastic pultruded components.

Material	Volume Fraction	Pultrusion Speed, m/min	Flexural Strength, MPa	Flexural Modulus, GPa	Tensile Strength, MPa	Elastic Modulus, GPa	Notched Izod Impact Strength, J/m^2^	Interlaminar Shear Strength, MPa	Reference
GF/Nylon 12	0.50	0.3–3.0	380–610	-	-	-	-	15–40	[162]
GF/Nylon 6	0.71–0.75	0.1–0.9	359–469	-	828–869	-	1868–2348	-	[199]
GF/ABS	0.75	0.1–0.9	538	-	710	-	-	-	[199]
GF/PPS	0.70–0.75	0.1–0.9	965	-	793	-	-	-	[199]
GF/PMMA	0.75	0.1–0.9	656–863	-	897–1035	-	1815–2188	-	[199]
GF/PBT	0.38–0.41	0.1–1.2	-	-	-	-	-	-	[144]
GF/PP	0.35	0.01–1.5	465–485	22–24	-	-	-	-	[94,95]
GF/PP	0.32	0.2–0.3	299–359	15–18	302–409	20–23	-	27–28	[192]
GF/PP	0.37	0.2–0.3	571–620	24–28	516–597	24–26	-	25–28	[192]
GF/PP	0.53–0.59	0.9	113–121	22	279–331	27–33	-	-	[194]
GF/PP	0.52	0.2–0.3	146–170	27–29	314–358	32–35	-	7–8	[192]
GF/PMMA	-	0.4	414	-	720	-	2400	-	[221]
GF/PMMA	-	0.7	207	-	530	-	1300	-	[221]
GF/PMMA	-	1.0	100	-	410	-	700	-	[221]
GF/PA 6 *	0.70	0.8	800–1060	26–34	-	-	-	61–70	[154]
GF/PU *	0.50	2.7	210	6	-	-	-	-	[222]
CF/PEEK	0.55	0.06–0.6	1150–1380	108–130	-	-	-	-	[143]
CF/Nylon 6	0.57	0.1–0.9	498	-	1496	-	1708	-	[199]
CF/PPS	0.58	0.1–0.9	1365	-	1172	-	1601	-	[199]
CF/PP	0.32	0.2–0.3	155–163	36–40	-	196–213	-	14	[192]
CF/PP	0.55	0.2–0.3	222–243	86–91	-	100–116	-	12–13	[192]
Flax/PLA	0.40	0.5–0.7	65–115	5–8	15–75	6–8	-	-	[99]
Graphite/PEI	0.61	0.18	1150	103	-	-	-	-	[196]
Graphite/PPS	0.61	0.08	1770	131	1820	117	-	-	[197]

* Reaction injection molding (RIM) pultrusion.

**Table 4:** Mechanical properties of thermoset pultruded components.

Material	Volume Fraction	Tensile Strength, MPa	Elastic Modulus, GPa
GF/Polyester	0.5–0.8	307–1320	21–59
GF/Vinylester	0.6	240	18–42
GF/Epoxy	0.5	414–790	32–40
CF/Vinylester	-	1400	140–145
CF/Epoxy	0.5–0.6	1213–2200	130–180
